# Correlation of Cell‐in‐Cell Structure With Prognosis in Solid Tumors—A Meta‐Analysis

**DOI:** 10.1155/bmri/4943372

**Published:** 2025-12-01

**Authors:** Haoyi Zi, Yinhai Dai, Mengxuan Li, Yidi Wang, Mao Wang, Shuai Wang, Yujie Bai, Jianing Sun, Cong Fan, Jiajun Ding, Ting Wang

**Affiliations:** ^1^ Shaanxi University of Chinese Medicine, Xianyang, Shaanxi, China, sntcm.edu.cn; ^2^ Department of Thyroid, Breast, and Vascular Surgery, Xijing Hospital of the Fourth Military Medical University, Xi′an, Shaanxi, China, fmmu.edu.cn; ^3^ Department of Surgical Oncology Medicine, The Second Affiliated Hospital of Shaanxi University of Chinese Medicine, Xianyang, Shaanxi, China

**Keywords:** cell-in-cell structure, prognosis, solid tumors

## Abstract

**Background:**

Cell‐in‐cell structures (CICs), a novel biomarker for complex cellular interactions, have garnered increasing attention for their potential in predicting cancer patient prognosis. However, the prognostic significance of CICs in tumor outcomes remains inconclusive. To address this, we conducted a meta‐analysis to assess the prognostic value of CICs in solid tumors, adhering to the Meta‐analyses Of Observational Studies in Epidemiology (MOOSE) guidelines.

**Methods:**

PubMed, Web of Science, and Cochrane Library databases were searched up to October 2024 for the retrieval of full articles. Studies related to the prognosis of cell‐in‐cell and solid tumors were considered eligible for analysis. The quality of the included studies was assessed according to the National Institute for Health and Clinical Excellence (NICE) Quality assessment tool.

**Results:**

We included 1836 patients with solid tumors to evaluate the association between overall cell‐in‐cell structures (oCICs) and prognosis, and 429 patients to evaluate the association between four subtypes of CICs (tumor‐in‐tumor [TiT], tumor‐in‐macrophage [TiM], macrophage‐in‐tumor [MiT], and lymphocyte‐in‐tumor [LiT]) and prognosis. We present the hazard ratio (HR) for overall survival (OS) for the number of CICs for each solid tumor. The combined HR for OS of oCICs was 1.64 (95% CI, 1.18–2.28; *p* = 0.003), and for LiT, it was 1.43 (95% CI, 1.12–1.83; *p* = 0.005), indicating that both oCICs and LiT are reliable prognostic factors for solid tumors. However, the combined HRs for OS of TiT, TiM, and MiT were 0.72 (95% CI, 0.35–1.48; *p* = 0.37), 1.28 (95% CI, 0.67–2.45; *p* = 0.46), and 1.54 (95% CI, 0.93–2.56; *p* = 0.09), respectively, suggesting that these subtypes may not be reliable prognostic factors due to the limited number of studies.

**Conclusion:**

The presence of higher numbers of oCICs and LiT is an adverse prognostic factor for patients and affects OS.

## 1. Introduction

Cell‐in‐cell structure (CIC) is an evolutionarily conserved biological phenomenon that is common in cells ranging from lower amoeba to higher mammalian cells, but has been reported in recent decades in human tumor cells, and is characterized by the existence of one or more structurally and functionally intact living cells inside another cell [1, 2]. This structure is generated by active cell‐cell interactions and is prevalent in a variety of human tumors, such as breast cancer [3–5], lung cancer [6], liver cancer [7], pancreatic cancer [8–11], and melanoma [12]. Studies have demonstrated that suppressing the expression of CDKN2A can significantly enhance the formation of homotypic CIC. As a tumor suppressor gene, inactivation of CDKN2A has been shown to facilitate homotypic CIC formation in human cancer cells [13]. Ruan et al. performed a microarray analysis to identify genes associated with homotypic CIC formation. Their work identified IL‐8 as a positive regulator of homotypic CIC formation via enhancing intercellular adhesion [14]. Many studies have shown that CICs can have an impact on tumor evolution, staging, and patient prognosis [15, 16]. In conclusion, an increasing amount of evidence substantiates the role of CICs as a prognostic marker for solid tumors, such as in early‐stage breast cancer [5], pancreatic cancer [10], and head and neck squamous cell carcinoma [17]. However, the prognostic effect of CICs on tumors has not reached a relatively consistent conclusion. Some studies have observed that CICs are associated with better prognosis. Cano et al. showed that CICs were negatively correlated with the incidence of pancreatic cancer metastasis [8]. Zhang et al. found that CICs are an independent protective factor for overall survival (OS) in patients with early‐stage breast cancer [5]. In contrast, other studies have suggested that CICs may predict a worse prognosis. Schwegler et al. found that the presence of CICs was an adverse prognostic factor for OS of patients with head and neck tumors [15]. Wang et al. found that CICs are a potentially poor prognostic marker for predicting survival in patients with hepatocellular carcinoma. Moreover, the presence of CICs was significantly associated with lower survival in patients with hepatocellular carcinoma [7]. Abodief et al. showed that the frequency of CICs in breast cancer was positively correlated with the malignancy of the tumor [3]. This was also confirmed by Krajcovic et al., with a higher rate of CIC formation in high‐grade breast cancer [18]. Ruan et al. have shown that a high frequency of CICs is associated with malignant progression in patients with advanced breast cancer [4]. The EML method established by Huang et al., which stains tumor samples with immunofluorescence, allows for a more detailed classification of CICs. Specifically, Huang et al. used E‐cadherin antibody to label epithelial cells, CD68 antibody to label macrophages, and CD45 to label white blood cells. This staining method is called the EML method and can specifically divide CICs into four subtypes. The four subtypes of CICs are tumor‐in‐tumor (TiT), tumor‐in‐macrophage (TiM), macrophage‐in‐tumor (MiT), and lymphocyte‐in‐tumor (LiT). TiM, MiT, and LiT are collectively known as heterotypic cell‐in‐cell structures (heCICs). In different tumors, CIC subtypes may be different due to TNM stage and tumor tissue inflammation, which also suggests the different roles of CIC subtypes in predicting tumor prognosis [19]. Su et al. demonstrated that by targeting regulatory molecules such as CD44 to enhance the formation of heterotypic CIC, intracellular killing and tumor growth could be effectively enhanced [20]. Wei et al. showed that heCICs were negatively correlated with the survival time of non‐small cell lung cancer (NSCLC) patients, suggesting that CIC subtypes could be used together with other classical clinicopathological factors to predict the prognosis of NSCLC patients [6]. Zhang et al. showed that prognostic analysis based on CIC subtypes is very necessary and contains more important information, and heterogeneous tumors usually produce different types of CICs, which may have different or even opposite effects on patient prognosis [5]. Thus, this meta‐analysis evaluated the prognostic value of CICs and their subtypes in solid tumors. Specifically, we systematically collated currently available data to further evaluate whether CICs and subtypes are reliable in predicting outcomes for patients with solid tumors.

## 2. Methods

PubMed, Web of Science, and Cochrane Library databases were searched up to October 2024 for retrieval of full articles. Studies related to the prognosis of cell‐in‐cell and solid tumors were considered eligible for analysis. The quality of the included studies was assessed according to the National Institute for Health and Clinical Excellence (NICE) Quality assessment tool. The protocol was registered on INPLASY (Registration Number: INPLASY2024100020). The study was designed according to the Meta‐analyses Of Observational Studies in Epidemiology (MOOSE) statement [21].

### 2.1. Inclusion Criteria

The study required survival analysis and CICs statistics, including extractable OS, hazard ratio (HR), overall cell‐in‐cell structures (oCICs), TiT, TiM, MiT, and LiT.

### 2.2. Exclusion Criteria

The exclusion criteria encompass the absence of access to the full text, a lack of pertinent data for the study, or insufficiently reported information; eligible article types include reviews, case reports, and conference abstracts.

### 2.3. Search Source and Term

Search for relevant articles in PubMed and Web of Science databases. An example of a retrieval strategy is the one used for PubMed: (((Cell‐in‐Cell Structure OR cytophagocytosis OR cell cannibalism OR entosis OR emperitosis)) AND ((tumor OR tumour OR cancer OR neoplasms))) AND ((prognosis OR prognostic)). The retrieved articles are then cross‐checked to ensure that all possible studies are retrieved.

### 2.4. Data Collection

The literature search process for the study was conducted independently by two investigators (H.Z. and J.D.). First, according to the title and abstract of the article, the relevance of the paper to this research is preliminarily judged, and irrelevant research papers are eliminated. Then, the two researchers independently evaluated the full text of the articles that passed the preliminary screening, eliminating those that did not conform to the study design or lacked relevant data. Any dispute between the two researchers regarding the relevance of an article is resolved by consensus. The data extraction process was performed by two investigators (H.Z. and J.D.) without interference, and the following information from the article was recorded: article title, first author, year of publication, number of included cases, study type, study design, oCICs, TiT, MiT, TiM, and LiT data.

### 2.5. Quality Evaluation of Included Studies

Study quality was assessed using the NICE Quality assessment tool [22].

### 2.6. Statistical Analysis

The statistics for each study were transcribed in tabular form. The study endpoint is OS. The effect size of HR was used to measure the impact of CICs on survival, with HR and 95% confidence intervals (CIs) extracted from each study. HR greater than 1 indicates poor survival in patients with high CICs, while HR less than 1 indicates a survival benefit in patients with high CICs. Heterogeneity among studies was assessed by the following method (I^2^). According to the Cochrane evaluator manual, I^2^ was evaluated as follows: I^2^ ≤ 40*%*, low heterogeneity; I^2^ ranged from 41% to 60% with moderate heterogeneity. I^2^ ranges from 61% to 100%, with significant heterogeneity [23]. For this reason, in oCICs, TiT, TiM, and MiT (I^2^ was 73%, 84%, 78%, and 61%, respectively), we used a random‐effects model. In LiT, since I^2^ = 45*%*, we used a fixed‐effect model. The meta‐analysis was conducted by Review Manager (RevMan, Version 5.3; Nordic Cochrane Centre, Cochrane Collaboration Network). Statistical significance was defined as *p* < 0.05.

## 3. Results

### 3.1. Search Strategy

**Figure 1:**
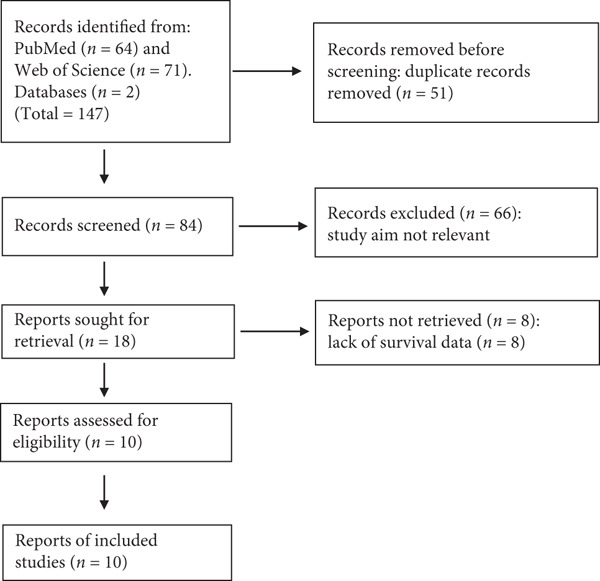
Flowchart of the selection process.

A total of 84 studies were retrieved after removing duplicates. By reading the title and abstract, 66 of these unrelated papers were excluded and the remaining 18 were included in the assessment. Finally, after screening using the above inclusion and exclusion criteria, a total of 10 studies were included in our meta score. Figure 1 shows the study flow chart, which indicates the reasons for excluding unqualified studies. The main features of the included studies are shown in Table 1.

**Table 1:** Main characteristics of the included studies.

**Study**	**Tumor type**	**Study design**	**Origin**	**Exposure**	**Effect size [95% CI]**	**p** **value**
Almangush A [24]	Oral tongue cancer	Retrospective cohort study	Europe	OCICs	2.99 [1.56, 5.74]	0.0010

Hayashi A 2020 [9]	Pancreatic ductal adenocarcinoma	Retrospective cohort study	America	oCICs	1.81 [1.32, 2.48]	0.0002

Huang H. 2020 [10]	Pancreatic ductal adenocarcinoma	Retrospective cohort study	Asia	oCICs	1.98 [1.32, 2.98]	0.0010
				TiT	1.65 [1.15, 2.36]	0.0067
				TiM	1.46 [0.90, 2.39]	0.1276
				MiT	1.97 [1.29, 3.00]	0.0016
				LiT	1.70 [1.24, 2.33]	0.0010

Schenker H. 2017 [16]	Head and neck squamous cell carcinomas	Retrospective cohort study	Europe	oCICs	1.78 [1.10, 2.89]	0.0197

Schwegler M. 2015 [15]	Head and neck squamous cell carcinomas Rectal cancer Anal carcinomas	Retrospective cohort study	Europe	oCICs	2.56 [1.33, 4.89]	0.0047

Song J. 2023 [11]	Pancreatic cancer	Retrospective cohort study	Asia	oCICs	2.50 [1.16, 5.37]	0.0188

Wang R. 2022 [7]	Hepatocellular carcinoma	Retrospective cohort study	Asia	oCICs	2.40 [1.36, 4.26]	0.0027

Wang Y. 2021 [25]	Esophageal squamous cell carcinoma	Retrospective cohort study	Asia	oCICs	1.56 [0.90, 2.71]	0.1099
				TiT	0.48 [0.25, 0.93]	0.0288
				TiM	1.18 [0.74, 1.87]	0.4944
				MiT	1.17 [0.75, 1.82]	0.4969
				LiT	1.03 [0.68, 1.56]	0.8984

Wei Y. 2023 [6]	Non‐small cell lung cancer (NSCLC)	Retrospective cohort study	Asia	oCICs	0.53 [0.23, 1.22]	0.1340
				TiT	0.54 [0.23, 1.25]	0.1508
				TiM	5.06 [1.56, 16.4]	0.0070
				MiT	0.25 [0.03, 1.79]	0.1680
				LiT	1.72 [0.54, 5.47]	0.3590

Zhang X. 2019 [5]	Breast cancer	Retrospective cohort study	Asia	oCICs	0.45 [0.25, 0.83]	0.0111
				TiT	0.53 [0.29, 0.98]	0.0414
				TiM	0.52 [0.29, 0.96]	0.0363
				MiT	2.41 [1.25, 4.66]	0.0090

Abbreviations: LiT, lymphocyte‐in‐tumor; MiT, macrophage‐in‐tumor; oCICs, overall cell‐in‐cell structures; TiM, tumor‐in‐macrophage; TiT, tumor‐in‐tumor.

### 3.2. Assessment of Methodological Quality and Bias Risk

NICE research quality assessment tool was used for evaluation. The quality evaluation results of included studies are shown in Table S1.

### 3.3. OS

**Figure 2:**
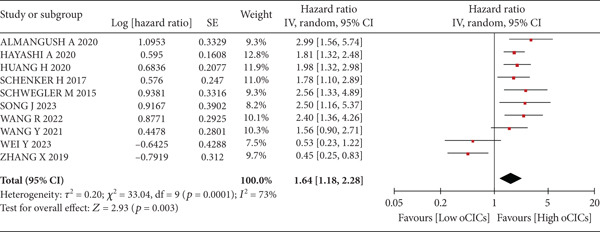
Forest plots of HRs for OS with oCICs.

**Figure 3:**
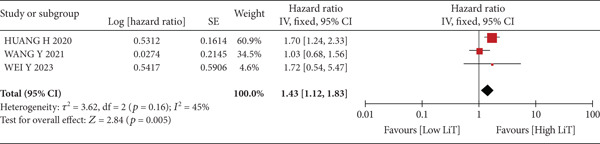
Forest plots of HRs for OS with LiT.

**Figure 4:**
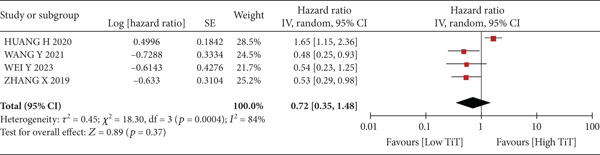
Forest plots of HRs for OS with TiT.

**Figure 5:**
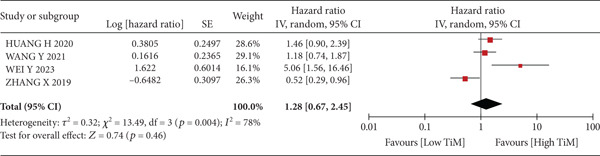
Forest plots of HRs for OS with TiM.

**Figure 6:**
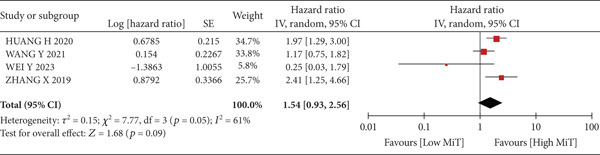
Forest plots of HRs for OS with MiT.

A total of 1836 patients were included to evaluate the relationship between oCICs and prognosis and 429 patients with solid tumors to evaluate the relationship between four subtypes of CICs and prognosis. The combined HR for OS of oCICs was 1.64 (95% CI, 1.18–2.28; *p* = 0.003) (Figure 2), LiT combined HR for OS was 1.43 (95% CI, 1.12–1.83; *p* = 0.005) (Figure 3). The combined HR for OS of TiT was 0.72 (95% CI, 0.35–1.48; *p* = 0.37) (Figure 4), the combined HR for OS of TiM was 1.28 (95% CI, 0.67–2.45; *p* = 0.46) (Figure 5), and the combined HR for OS of MiT was 1.54 (95% CI, 0.93–2.56; *p* = 0.09) (Figure 6). The funnel plot, displayed in Figure 7, did not suggest that there was publication bias. In conclusion, both oCICs and LiT can be used as relatively reliable factors to predict the prognosis of solid tumors.

Figure 7Funnel plot for publication bias.

**Figure:**
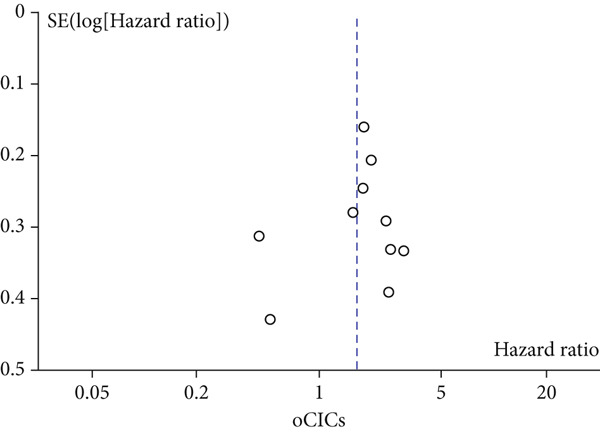
(a)

**Figure:**
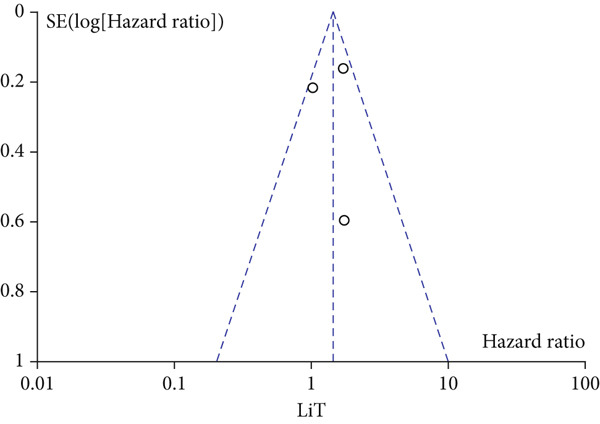
(b)

**Figure:**
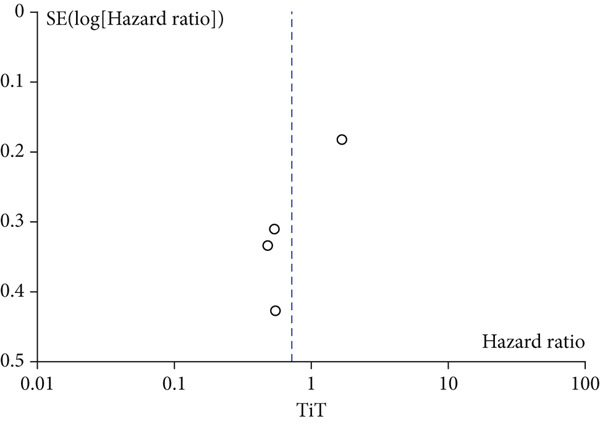
(c)

**Figure:**
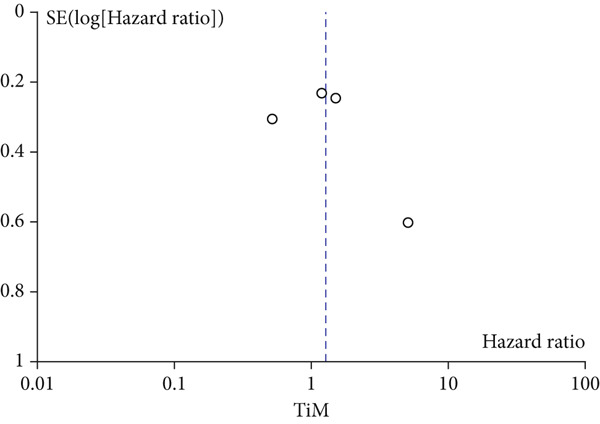
(d)

**Figure:**
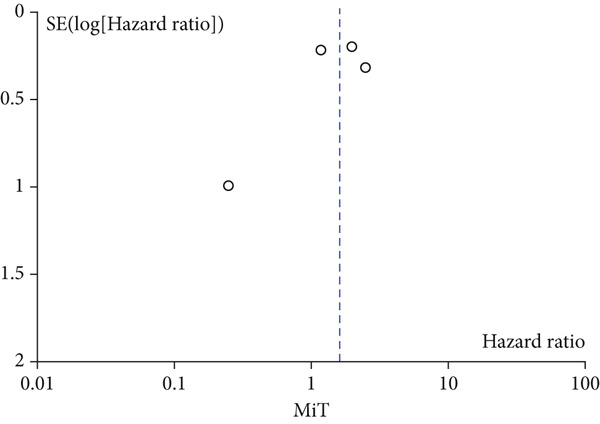
(e)

## 4. Discussion

CICs were found in both normal epithelial cells and tumor tissue, but appeared to be more common in tumor tissue [26]. In various types of tumors, including breast cancer, lung cancer, liver cancer, pancreatic cancer, and esophageal cancer, CICs were believed to be strongly correlated with prognosis. Due to the lack of high‐quality randomized controlled trials, the relationship between CICs and prognosis in patients with solid tumors cannot be drawn a relatively clear conclusion. We comprehend that this is the inaugural meta‐analysis to explore the prognostic significance of CICs in solid tumors. Our findings imply that oCICs and LiT are dependable predictors of OS and can be employed as prognostic indicators for solid tumors. Wei et al. showed that in lung cancer, more CICs could be found in adenocarcinoma tissues compared to squamous cell carcinoma. LiT was more common in the upper lobe of the lung than in other lobes. The presence of TiM and heCIC was highly correlated with OS in patients with NSCLC [6]. Wang et al. showed that the presence of CICs was significantly correlated with the low survival rate of HCC patients, and multivariate Cox regression analysis further confirmed that CICs were an independent risk factor for poor survival (HR = 1.902, *p* = 0.047) [7]. Bauer et al. found that patients with CIC positive had a poorer prognosis for metastasis‐free survival (MFS). Subgroup analysis showed that there was a correlation between high proliferation index and high CIC rate [27]; this suggested that CICs may affect the ability of tumors to proliferate. Wang et al. showed that TiT was an independent prognostic factor for resectable ESCC, and patients with higher TiT density had longer postoperative survival [25]. Zhang et al. also found that the higher TiT, the longer the survival of patients (HR = 0.529, 95% CI, 0.288–0.973; *p* = 0.04) [5]. This was consistent with our findings that TiT was more likely to be an independent protective factor. Perhaps due to the small number of TIT‐related studies, our results showed no statistical significance between TiT and OS due to the heterogeneity among studies (HR = 0.72 (95% CI, 0.35–1.48; *p* = 0.37)). Similarly, we believed that TiM and MiT may also have no statistical significance with OS due to the same reason (TiM, HR = 1.28 (95% CI, 0.67–2.45; *p* = 0.46), MiT, HR = 1.54 (95% CI, 0.93–2.56; *p* = 0.09)). Overall, our results reaffirmed the association of CICs with tumor prognosis. Secondly, the relationship between CICs subtypes and tumor prognosis also provided us with a broader idea to understand the impact of various cell interactions in the tumor microenvironment on tumors. In addition, the consequences of interactions between immune cells and tumor cells can be further elucidated by CICs subtypes such as LiT, MiT, and TiM. However, this meta‐analysis had some limitations, mainly due to the fact that all the included studies were retrospective studies. Secondly, there are relatively few studies related to CICs, and we only included 10 relevant studies. Therefore, further randomized controlled trials were needed to better evaluate the prognostic role of CICs and its subtypes in solid tumors. In addition, our study found that patients with high oCICs and LiT had a higher risk of death compared to patients with low oCICs and LiT, and existing studies did not have an optimal threshold to accurately classify the number of CICs. Future studies may be able to fill this gap, allowing CICs to be accurately classified and further clarify their role in tumor prognosis.

## 5. Conclusions

We included 1836 patients with solid tumors to assess the relationship between oCICs and prognosis and 429 patients with solid tumors to assess the relationship between four subtypes of CICs and prognosis. The results showed that oCICs were a predictor of OS (HR = 1.64 (95% CI, 1.18–2.28; *p* = 0.003)) and could be utilized as a prognostic factor for solid tumors. Similarly, the results of TiT, TiM, MiT, and LiT studies on 429 patients with solid tumors showed that LiT was a predictor of OS (HR = 1.43 (95% CI, 1.12–1.83; *p* = 0.005)), can be used as a prognostic factor for solid tumors.

## Disclosure

All authors have read and agreed to the published version of the manuscript.

## Conflicts of Interest

The authors declare no conflicts of interest.

## Author Contributions

Conceptualization: H.Z. and Y.D. Methodology and formal analysis: H.Z., J.D., M.L., S.W., and Y.B. Data curation: H.Z., J.D., Y.D., M.W., J.S., and C.F. Writing—original draft preparation: H.Z. and J.D. Writing—review and editing: T.W. Visualization: H.Z. and J.D.

## Funding

This study is supported by the Cultivation Boost Project of Xijing Hospital (No. XJZT24LY09) and the Natural Science Basic Research Program of Shaanxi Province (10.13039/501100017596; No. 2021JZ‐29 and No. 2023‐JC‐QN‐0965).

## Supporting information


**Supporting Information** Additional supporting information can be found online in the Supporting Information section. Table S1: The National Institute for Health and Clinical Excellence’s Case Series Study Quality Assessment checklist. Authors are responsible for providing the final supporting information material files that will be published along with the article.

## Data Availability

The data that support the findings of this study are available from the corresponding author upon reasonable request.
