# Supplementary material for: Correlation of Cell‐in‐Cell Structure With Prognosis in Solid Tumors—A Meta‐Analysis
Source: Biomed Res Int. 2025 Dec 1;2025:4943372. doi: 10.1155/bmri/4943372 (PMC12666160; doi:10.1155/bmri/4943372)
Supplement: Supplementary file 1 — Supporting Information Additional supporting information can be found online in the Supporting Information section. Table S1: The National Institute for Health and Clinical Excellence’s Case Series Study Quality Assessment checklist. Authors are responsible for providing the final supporting information material files that will be published along with the article. [file BMRI-2025-4943372-s001.zip › Table S1.docx]

**Table S1.Quality Assessment of case series studies check list from National Institute for Health and Clinical Excellence.**

| **Study** | **Multicenter** | **Aim** | **Inclusion/Exclusion Criteria** | **Outcome** | **Prospective** | **Consecutive** | **Main Findings** | **Stratified** |
| --- | --- | --- | --- | --- | --- | --- | --- | --- |
| ALMANGUSH.A 2020 | No | Yes | Yes | Yes | No | Yes | Yes | No |
| HAYASHI.A 2020 | No | Yes | Yes | Yes | No | Yes | Yes | No |
| HUANG.H 2020 | No | Yes | Yes | Yes | No | Yes | Yes | No |
| SCHENKER.H 2017 | No | Yes | Yes | Yes | No | Yes | Yes | No |
| SCHWEGLER.M 2015 | No | Yes | Yes | Yes | No | Yes | Yes | No |
| SONG.J 2023 | No | Yes | Yes | Yes | No | Yes | Yes | No |
| WANG.R 2022 | No | Yes | Yes | Yes | No | Yes | Yes | No |
| WANG.Y 2021 | No | Yes | Yes | Yes | No | Yes | Yes | No |
| WEI.Y 2023 | No | Yes | Yes | Yes | No | Yes | Yes | No |
| ZHANG.X 2019 | No | Yes | Yes | Yes | No | Yes | Yes | No |
